# Acute Inflammatory Demyelinating Polyradiculoneuropathy‐like Associated with Subcutaneous Foslevodopa–Foscarbidopa: First Report

**DOI:** 10.1002/mds.70241

**Published:** 2026-02-26

**Authors:** Heloise Cochez, Corentin Cazenave, Celestin Castagnet, Laure Balguerie, Blandine Acket, Margherita Fabbri, Fabienne Ory‐Magne

**Affiliations:** ^1^ Department of Neurology Toulouse University Hospital Toulouse France; ^2^ Department of Clinical Pharmacology Toulouse Parkinson Expert Centre, Toulouse NeuroToul Center of Excellence in Neurodegeneration (COEN), French NS Park/F‐CRIN Network, University of Toulouse 3, CHU of Toulouse, INSERM Toulouse France

**Keywords:** acute polyneuropathy, adverse events, EMG, Parkinson's disease

Continuous subcutaneous infusion of foslevodopa–foscarbidopa (LDp/CDp) is a recent addition to the therapeutic armamentarium for managing motor fluctuations in Parkinson's disease (PD). We report a case of acute inflammatory demyelinating polyradiculoneuropathy‐like (AIDP‐like) occurring during LDp/CDp therapy. Although not previously described with LDp/CDp, comparable acute neuropathies have been reported with levodopa‐carbidopa intestinal gel (LCIG), indicating a potential effect of high‐dose levodopa delivery.

## Case Report

The patient, diagnosed with akinetic‐rigid PD at 51 years of age, had a medical history notable for antiphospholipid syndrome with multiple thrombotic events. He repeatedly declined deep brain stimulation. For 4 years, he remained clinically well controlled on continuous subcutaneous apomorphine infusion (APO) before being admitted in June 2025 (age, 64 years) for a switch to LDp/CDp because of persistent on/off fluctuations and disabling dyskinesias. Baseline clinical and laboratory data are presented in Table S1. Neurological examination showed normal reflexes and intact sensation, except for slightly reduced vibration sense at the medial malleoli (7 seconds). He declined pre‐treatment nerve conduction studies. After a 1‐week titration of LDp/CDp (0.9 mL/hr during the day and 0.30 mL/hr overnight), his condition improved.

During the week after discharge, transient hallucinations and dyskinesias resolved after reducing ropinirole. Two months later, he reported weight loss, no dyskinesias, but reduced behavioral ON and mild slowness, which led to an increase in daytime LDp/CDp and a gradual taper of amantadine. Fifteen days before a scheduled hospitalization (planned 4 months after starting LDp/CDp), he developed acute distal paresthesia and worsening gait, although he became bedridden he did not seek urgent care. At admission, he alternated between normal cognition and brief episodes of syncopal events. Neurological examination showed dorsiflexor and extensor weakness, global areflexia, profound glove‐and‐stocking sensory loss, and severe cardiovascular dysautonomia (pronounced postural heart rate and blood pressure variability). Electroneuromyography demonstrated non length dependent sensory impairment, multifocal conduction block on both ulnar and median nerves and prolonged F latencies consistent with AIDP. There was no resting activity, and motor unit potential (MUP) recruitment was depleted, consistent with demyelinating damage on needle detection test. No alternative cause was identified, including vaccination, recent infection, or any other pathology as described in Table S1. However, a lumbar puncture was not performed in the absence of an infectious context and because the patient was on anticoagulants at a therapeutic dose, with unstable INR control.

LDp/CDp was discontinued, and the patient received intravenous immunoglobulin (2 g/kg over 5 days) together with B6, B9, and B12 supplementation, and oral levodopa was resumed. Over the following 2 weeks, he showed partial recovery of distal strength and resolution of dysautonomia.

## Discussion

Although AIDP has not previously been reported with LDp/CDp, its occurrence aligns with concerns raised from LCIG experience. Multiple studies have documented peripheral neuropathies, including severe demyelinating polyradiculoneuropathies resembling AIDP‐like under LCIG therapy.^1^, ^2^, ^3^, ^4^ Mechanisms implicated in LCIG‐associated neuropathy are likely relevant to LDp/CDp, and to date, one acute polyneuropathy under LDp/CDp has been described.^5^ Transitioning from APO to LDp/CDp markedly increased this patient's levodopa exposure, a recognized risk factor. High levodopa intake increases S‐adenosyl‐methionine utilization during COMT‐mediated methylation, thereby lowering vitamins B6 and B12 and raising homocysteine and methylmalonic acid metabolic disturbances known to induce oxidative stress, inflammation, and impaired DNA repair.^6^ Additional risk factors described under LCIG such as weight loss,^4^ pre‐existing PD‐related large‐fiber neuropathy, and genetic susceptibility (MTHFR C677T, COMT Val158Met) may further modulate individual vulnerability.^7^

This case demonstrates that acute AIDP‐like with LDp/CDp, although novel, is not entirely unexpected. It represents the clinical expression of mechanisms already recognized in LCIG‐treated patients. As LDp/CDp use expands, systematic metabolic monitoring, vitamin supplementation, and vigilant screening for early neurological symptoms may be essential to reduce the risk of severe neuropathy.

## Author Roles

(1) Research project: A. Conception, B. Organization, C. Execution; (2) Statistical Analysis: A. Design, B. Execution, C. Review and Critique; (3) Manuscript Preparation: A. Writing of the first draft, B. Review and Critique:

H.C.: 3A

Co.Ca: 3B

Ce.Ca: 3B

L.B.: 3B

B.A.: 1C, 3B

M.F.: 3B

F.O.M.: 1C, 3B

## Financial Disclosures for the Previous 12 Months

FOM has served as an advisory board member or consultant or has received travel grants from AbbVie, Aguettant, Orphalan, Orkyn, Biogen, Adelia, NHC France, and Convatec. She reports grants from Fondation Santé Service. MF has served as an advisory board member or consultant from Bial and Orkyn. She received honoraria to speak from Bial, Orkyn, AbbVie, and Everpharma.

## Financial Disclosures and Conflicts of Interest

No specific funding was received for this work. The authors declare that there are no conflicts of interest relevant to this work. Author disclosures are available in the S1.

## Supporting information


**Data S1.** Supporting Information.
