# Supplementary material for: Acute Inflammatory Demyelinating Polyradiculoneuropathy‐like Associated with Subcutaneous Foslevodopa–Foscarbidopa: First Report
Source: Mov Disord. 2026 Feb 26;41(5):1317–8. doi: 10.1002/mds.70241 (PMC13206383; doi:10.1002/mds.70241)
Supplement: Supplementary file 1 — Data S1. Supporting Information. [file MDS-41-1317-s001.zip › Table 1 sans modif.pdf]

Table 1. Clinical characteristics and outcomes of the patient at initiation (admission and discharge) and AIDP-like occurrence.

Baseline clinical and laboratory data	Initiation		AIDP-like occurence
	Admission	At discharge	
**BMI** *(kg/m²)*	27.7	-	24.9
**MMSE** *(/30)*	30	-	-
**Score MDS-UPDRS**			
MDS-UPDRS III MED OFF	61	-	-
MDS-UPDRS III MED ON	10	-	40
Hoehn & Yahr MED OFF	5	-	5
Hoehn & Yahr MED ON	3	-	4
MDS-UPDRS IV A	3	-	0
MDS-UPDRS IV B	8	-	6
**OFF duration** *(hours)*	6.3	-	4
**ON duration without troublesome dyskinesia** *(hours)*	1.7	-	0
**ON duration with troublesome dyskinesia** *(hours)*	3.7	-	0
**Antiparkisonian treatments**			
Subcutaneous infusion of apomorphine, *LEDD (mg)*	*1225*	-	-
Subcutaneous infusion of LDp/CDp, *LEDD (mg)*	-	0.9ml/h day-time 0.3ml/h overnight, *2987*	0.96ml/h day-time 0.3ml/h overnight, *3167*
Amantadine (mg)	300	200	-
Ropinirole (mg), *LEDD (mg)*	8 *(160)*	8 *(160)*	4 *(80)*
Levodopa–Benserazide (mg)	950	150	200
LEDD total (mg)	2635	3497	3447
**Comorbidities**			
High blood pressure			
Antiphospholipid antibody syndrome			
Hypercholesterolemia			
Benign prostatic hyperplasia			
**Associated treatments**			
Lercanidipine chlorhydrate 10mg	+	+	+
Hydroxychloroquine sulfate 200 mg	+	+	+
Silodosine 8 mg	+	+	+
Simvastatine 40 mg	+	+	+
Folate and vitamin B12 supplementation	-	+	+
**Biology**			
Vitamin B1 (nmol/l) *normal range 91-153*	149	-	189
Vitamin B6 (nmol/l) *normal range 51-183*	53	-	26
Folate (ng/ml) *normal range > 5.38*	4	-	12.6
Vitamine B12 (pg/ml) *normal range 197-771*	612	-	1224
Homocysteine (µmol /l) *normal range <15*	25.9	-	83
Serum protein electrophoresis	-	-	normal
Serum immunofixation	-	-	absence

Anti-ganglioside antibodies	-	-	negatif
Infectious serology (EBV, CMV,	-	-	negatif
Campylobacter jejuni, Haemophilus influenzae,	-	-	negatif
HIV, HBV, HCV, Lyme, Syphilis)	-	-	negatif
Hepatisis E serology	-	-	IgM-/ IgG +
Anti-nuclear antibodies	-	-	negatif
Onconeural antibodies	-	-	negatif
Neurofilament light (pg/mL) *normal range 6-23*	-	-	2023

BMI, Body Mass Index; LEDD, L-dopa equivalent daily-dose; MMSE, Mini-Mental State Examination, score range 0-30.
